# Comparison between different intermaxillary fixation systems in the surgical and orthopedic treatment of non-condylar mandibular fractures. Systematic review

**DOI:** 10.4317/medoral.27264

**Published:** 2025-05-27

**Authors:** María José Flores Mancilla, Marcelo Arqueros Lemus, Pedro Tapia Contreras

**Affiliations:** 1 Dental Surgeon, Andrés Bello University, Viña del Mar, Chile; 2 Resident in Oral and Maxillofacial Surgery, University of Chile, Santiago, Chile; 3 Maxillofacial Surgeon, Franco Ravera Z. Hospital, Rancagua, Chile; 4 Maxillofacial Surgeon, Maxillofacial Surgery Department, Clínica Red Salud Vitacura, Santiago, Chile

## Abstract

**Background:**

The treatment of mandibular fractures can be orthopedic and/or surgical; in both modalities, intermaxillary fixation is a therapeutic tool that allows for the stabilization and reduction of fractures, guiding dental occlusion There are different methods of intermaxillary fixation, each with individual characteristics that provide therapeutic options for the practitioner. This study aims to perform a quantitative and qualitative comparison of different features of these intermaxillary fixation systems through a systematic review.

**Material and Methods:**

A systematic review was performed, following the PRISMA guidelines. The Pubmed, SCOPUS, Web of Science and Cochrane databases were searched. Several variables were considered and are presented comprehensively in Tables and Figures. The initial literature search resulted in 51 articles, of which 9 met the inclusion criteria for the analysis.

**Results:**

Of the 51 identified articles, 28 were analyzed, with 19 excluded after full-text evaluation. Ultimately, 9 studies with 3,221 patients were included, comparing Erich arch bars (EAB), hybrid arch bars (HAB), and intermaxillary fixation screws (IMFS). Discussion: The studies focused on simple fractures with sufficient teeth for orthopedic treatment, excluding isolated maxillary fractures. Results showed differences in installation time, occlusal stability, oral hygiene, and costs, with EAB being the most expensive. Patient quality of life and complications, such as screw loss and root perforations, were also evaluated. Most studies presented a low risk of bias.

**Conclusions:**

Screw-based methods like IMFS and HAB offer shorter installation times than EAB, reducing surgery duration, costs, and biosecurity risks. While EAB remains a valid option, screw methods provide advantages in time, hygiene control, and biosecurity, with the choice depending on patient needs and surgical experience.

** Key words:**Erich bars, mandibular fractures, screw fixation.

## Introduction

Maxillofacial trauma is one of the most significant health problems worldwide and increasingly concerning for public health due to its high morbidity and mortality rates (1). Due to its extension and anatomy, the mandible is one of the most affected anatomical regions by this type of trauma (33%)(2). Successful treatment depends on the reduction of fractured segments to their normal anatomical positions and the prevention of movement through the fixation of bone segments, either by an open or closed technique. In both, the commonly used method for fracture reduction is intermaxillary fixation (IMF) (3,4).

Currently, multiple IMF techniques have been described, Erich arch bars (EAB) is the most used intermaxillary fixation screws (IMFS) method due to its low cost and occlusal reproducibility but has drawbacks like long installation time and injury risks (5-8). IMFS offers faster procedures and better patient comfort but is more expensive and carries risks like root injury. Hybrid arch bars (HAB), combines EAB with screw fixation, providing easier application, improved comfort, and better oral hygiene maintenance. (9-11)

Favorable handling properties and low complication rates have been reported using HAB; indeed, studies report benefits in installation time compared to EAB (7). However, there is insufficient scientific evidence to prove complete superiority of these methods over the conventional method of fixation using EAB. Therefore, our objective is to conduct a systematic review comparing perioperative and postoperative parameters of using EAB with fixation methods using screws, whether IMFS and/or HAB, in patients with mandibular fractures.

## Material and Methods

The following review was conducted following the recommendations outlined in PRISMA-ScR (12) (Preferred Reporting Items for Systematic Reviews and Meta-Analyses) to perform a systematic review regarding a comparative evaluation between the use of EAB as a conventional IMF method and fixation methods utilizing screws, whether with IMFS and/or HAB, in the management of mandibular fractures. The research question for this study was formulated using the PICO guidelines (Population, Intervention, Control, Outcome) as follows: In patients with mandibular fractures requiring IMF (P), is the use of EAB the most effective and cost-efficient IMF method compared to fixation methods using screws (IMFS and/or HAB), when evaluating installation time, stability, patient tolerance, oral hygiene, cost, and complications?

- Eligibility Criteria

The eligibility criteria used in selecting primary studies were as follows: Full articles in English published between 2019 and 2024 that compare the use of EAB with fixation methods utilizing screws, whether with IMFS and/or HAB as IMF methods in patients with mandibular fractures treated orthopedically and/or surgically. Additionally, cohort studies and randomized or non-randomized clinical studies were included. Studies reporting pathological mandibular fractures due to odontogenic tumors or bone metabolism disorders and studies conducted on animals, as well as manuscripts, books, letters to the editor, opinions, and reviews, were excluded.

- Sources Of Information

To identify potentially relevant articles, the following bibliographic databases were selected: PubMed, Scopus, Web of Science, and Cochrane Library. A search was conducted independently by authors (M.F. and M.A.) between May 1 and June 4, 2024.

- Search Strategy

According to the described protocol, an electronic search was conducted based on the selected databases according to the research question. The search terms used were: “Erich arch bar,” “Mandibular fractures,” “Intermaxillary screws,” and “Hybrid arch bars,” which were combined with Boolean operators “AND,” “OR,” and "NOT." A search strategy was developed for each database as appropriate. From the obtained results, a manual search for additional potential articles was executed by reviewing the reference list of the identified primary articles.

- Evidence Selection

Evidence selection was conducted independently by two reviewers (M.F. and M.A.). The primary data from the initially identified articles were exported to an Excel spreadsheet. The two reviewers independently analyzed the titles and abstracts and identified articles eligible for a full review. Selected articles were imported into the Mendeley bibliographic citation manager (Mendeley Ltd, London, UK) to facilitate their organization. Subsequently, a full-text review of each selected article was conducted, and those meeting the eligibility criteria were included. Disagreements were resolved by a third author (P.T.).

- Data Extraction

For the collection and extraction of data from each of the included studies, the following was considered: author, year, country of origin, study design, sample, IMF method, procedure duration, occlusal stability, oral hygiene, costs, and associated complications.

- Bias Risk Assessment

To evaluate the quality of evidence from the selected articles, the Joanna Briggs Institute (JBI) critical appraisal tool for randomized controlled trials and cohort studies was used. For randomized controlled trials, this tool evaluates thirteen domains, considering studies that scored between 13 and 10 as low risk of bias, those scoring between 9 and 5 as medium risk, and those scoring 4 points or less as high risk of bias. For the included cohort studies, eleven domains were evaluated, considering studies scoring between 11 and 10 points as low risk of bias, those scoring between 9 and 8 points as medium risk, and those scoring 7 points or less as high risk of bias.

## Results

**Figure 1:**
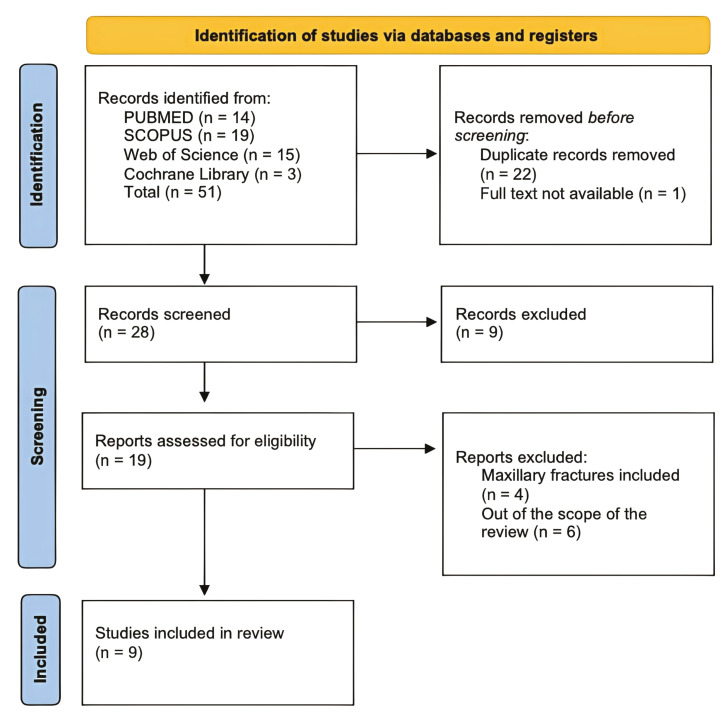
PRISMA Flow diagram.

The systematic search yielded a total of 51 articles for review. After eliminating duplicates, 28 articles were selected for title and abstract analysis. Of these, the full text of 28 was analyzed, and 19 studies were discarded for various eligibility criteria. Finally, 9 articles met the inclusion criteria and were included for data extraction (Fig. 1).

Regarding study designs, 3 studies were cohort studies (13-15) and 6 were randomized controlled trials (7,15-20). Of the nine included studies, 55.55% (7,15,18-20) were from the East, 33.33% (14,15,17) from North America, and 11.11% (16) from South America. These included a total of 3,221 patients with an average age of 31.62 years (range 16-60 years), predominantly male (7,13-20) (Table 1).

Seven of the included studies compared the use of EAB and HAB (7,13,15,17-20), one study compared the use of EAB and IMFS (16), while another study compared the use of all three devices (14). The sample of patients with EAB was 296, HAB was 193 (7,13-20), and IMFS was 51 (14,16).

- Characteristics Of Results

Isolated maxillary fractures, bimaxillary, condylar, comminuted mandibular fractures, or fractures older than 2 weeks were excluded. Simple, uni or bilateral fractures in the angle, body, parasymphysis, or symphysis with sufficient teeth for orthopedic treatment were included (7,13-20).

- Characteristics Of Arches And Screws

Nine studies reported the characteristics of arches and screws (7,13-20). Stainless steel wires of 0.4-0.5mm were used (7,15,17), with the arch generally positioned from the first molar to the first molar on the opposite side (16,17,19). Regarding the screws, 2, 4, 5, or 6 units per arch were used (7,14-20), with diameters ranging from 2 x 6 to 2 x 9 mm (7,16,19) or 1.5 x 6 to 1.5 x 8 mm (15,18,20) (Table 1).

- Installation Time

Regarding the installation time of the apparatus, it was compared across all articles (7,13-20), except one (20) which did not provide further information in its research. The average installation time for EAB was 82.37 minutes, followed by the installation of HAB with an average of 49 minutes (7,13-20), and studies analyzing the installation time of IMFS reported an average of 9 minutes (14,16) (Table 1).

- Occlusal Stability

Of the 9 studies, 7 compared occlusal stability (7,15-19), which was measured by the operator, excluding dislodgement and breakage of arches, wire loss, and screw loss. In 2 studies, wire loss in EAB arches was reported: Venugopalan *et al*. recorded the loss of one wire, and Sankar *et al*. reported that one patient lost two wires; however, this did not affect occlusal stability. The arches were not removed until the 4th or 6th week of orthopedic treatment (7,14-19).

- Oral Hygiene

Seven studies (7,15-19) compared oral hygiene using the Visible Plaque Index (VPI). This index is a clinical tool that measures the presence of visible dental plaque on teeth. The VPI classifies oral hygiene into different categories, such as "good," "fair," or "poor," based on the amount of visible plaque during the clinical examination. In these studies, results showed similar indices between the EAB and HAB groups, mostly classified as "good" or "fair." However, in four specific studies (7,15,16,20), there was less biofilm control in the EAB group, indicating a greater presence of visible plaque compared to the HAB group. Fernandes *et al*. (16) evaluated the VPI and the Gingival Bleeding Index (GBI) comparing EAB with IMFS, obtaining a higher visible plaque index in the first group than in the second; however, for GBI, no significant differences were observed.

- Cost

Two studies discussed cost in their results. One study (14) reported higher costs for EAB, followed by HAB and subsequently IMFS, where the latter two decreased by 34% and 15%, respectively, from the total cost of EAB when analyzing installation, materials, and removal operating expenses. When secondary procedure costs were included, Erich arch bars remained the most expensive. The second study (13) calculated the cost in dollars/minute evaluating arch/screw cost, anesthesia, and personnel. It revealed that the use of HAB reduces costs compared to EAB only when operating room fees are high. In unilateral fractures, the treatment cost comparing HAB with EAB is reduced by 4%, and in bilateral fractures by 11%. This implies that HAB is efficient in cost reduction only when these are sufficient to justify the investment in the product. Moreover, the cost that HAB adds to surgery decreases as operating room fees and surgical time increase.

- Quality Of Life

Fernandes *et al*. evaluated the quality of life of patients undergoing EAB and HAB using a questionnaire (16). They found that most patients subjected to EAB felt "embarrassed," followed by "feeling less satisfied with life," and lastly "painful." Merna *et al*. compared postoperative pain in both groups, being less painful in the first week in the HAB group; however, by the sixth week, greater pain was reported in the EAB group. The rest of the studies (7,13-15,17-20) did not report quality of life evaluation results in patients undergoing IMF.

- Complications

Screw loss was reported in one study (7). Three articles reported results of root perforation (7,14,15), while one of the articles with screws reported no complications. (8). Four of them reported mucosal overgrowth over them (7,16,19,20). Concerning biosecurity, 6 studies reported needlestick accidents or glove perforation during EAB installation (7,14,16,17,19,20) (Table 1).

- Bias Risk Assessment

**Figure 2:**
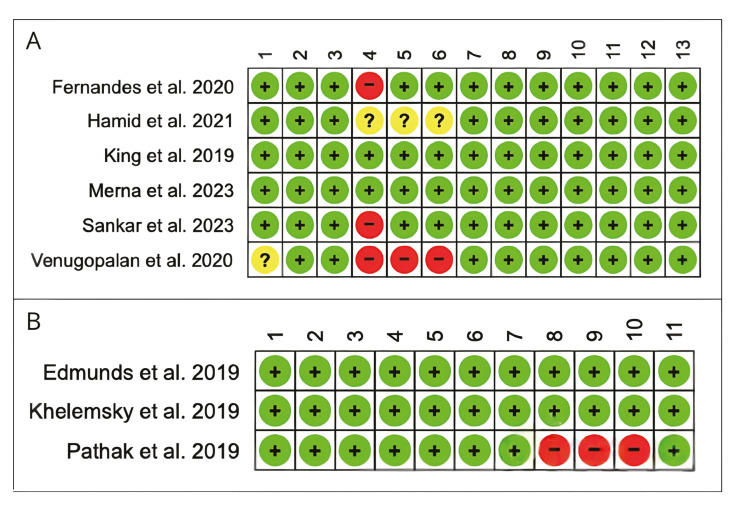
(A) Risk of bias in randomized controlled trials, (B) Cohort studies.

The results obtained through the application of the JBI critical appraisal tools for randomized controlled trials and cohort studies are detailed in Fig. 2. Regarding randomized controlled trials, 5 studies were classified as low risk of bias (7,16-20), and 1 study as moderate risk of bias due to not describing the study's randomization steps (18). In the cohort studies, all studies presented a low risk of bias (13,14), except one that was considered to have a moderate risk of bias due to not reporting long-term follow-up in the studied patients (15).

## Discussion

EAB has been considered a conventional method and the most used to date; however, there are several disadvantages associated with its use (7). Due to the various current IMF alternatives, the purpose of this systematic review is to compare perioperative and postoperative parameters of using EAB with fixation methods using screws, whether IMFS and/or HAB, in patients with non-condylar mandibular fractures.

Our systematic review found that EAB has a significantly longer installation time (82.37 minutes) (7,13-20) compared to IMFS (9 minutes) (14,16). This is due to the complex wiring process required for EAB, whereas screw-based systems need fewer instruments and installation sites, making them a faster and more efficient option for IMF.

Despite their advantages, screws can lead to complications such as mucosal coverage (7,16,19,20)., which can cause discomfort and pain. Studies report that by the fourth week, screws are often partially or completely covered, with improper placement near the vestibule increasing this risk. (16,19). Fernandes *et al*. (16) state that to avoid mucosal coverage, it must be ensured that screws are not placed too close to the vestibule and that they are located coronally, at the limit between the attached gingiva and mucosa, to prevent local trauma that could lead to mucosal overgrowth over the screw (21).

Root perforation mainly occurs in lower incisors due to limited space, so screw placement is recommended in the premolar region to avoid complications. Screw loosening was reported previously in 17% of cases but did not significantly affect device stability (1). While screw-based methods have risks, proper placement techniques can reduce complications. In contrast, EAB poses biosecurity risks, such as needlestick injuries and glove perforations, which studies suggest can be minimized by using double gloves (22-25).

Regarding oral hygiene, EAB leads to greater biofilm accumulation due to its complex wiring, making oral hygiene difficult. HAB improves hygiene by reducing wire extensions, while IMFS minimizes biofilm the most by eliminating devices near the teeth, promoting faster healing and reducing infection risk (7,15-19,26

When evaluating stability, the following study reported no differences in occlusal stability between EAB and HAB/IMFS groups. These findings align with previous studies like Rai *et al*. (6). While certain studies refer to loosening of arches and/or wires (3,4,8), they indicate that these do not interfere with final stability after follow-up extending up to 6 weeks in orthopedic/orthopedic-surgical treatments. No significant differences in final occlusal stability were found between EAB and screw-based methods (7,13,17-20). This suggests that both approaches are effective in maintaining occlusal functionality after the treatment of mandibular fractures.

Khelemsky *et al*. (13) propose that IMF with HAB and IMFS is cost-effective when comparing values assigned by surgical facilities, as it is an easier method to remove, thus shortening the stay in the operating room, allowing for additional surgeries within a fixed assigned surgical time. Edmunds *et al*. (14) provides dollar values associated with the cost of necessary materials, anesthesia, physical space, and operating rooms. Regarding materials, the most expensive technique is HAB, followed by IMFS and EAB; however, when adding the cost of operating rooms, like Khelemsky (13), the EAB technique remains less profiTable due to the time required for the removal of the apparatus, regardless of whether it is unilateral or bilateral fractures. The importance of this analysis is that HAB is "efficient" in minimizing costs only when the total accumulated costs of surgery are sufficient to compensate for the investment in the product. Although EAB is the least expensive method in terms of materials, its greater installation and removal time make it less profiTable in contexts where operating room costs are high. In contrast, HAB and IMFS offer a favorable cost-benefit ratio in these scenarios, being more efficient in surgeries requiring reduced surgical times.

A limitation of the study lies in the heterogeneity of the methods and evaluated variables: differences in materials, screw sizes, wire gauges, and installation techniques influence the results, particularly regarding occlusal stability and complications. Additionally, the reliance on subjective measurements performed by operators and the lack of quantitative evaluation methods introduces possible biases. More randomized studies and longer follow-ups are suggested to reduce bias.

## Conclusions

Screw-based methods, particularly IMFS and HAB, offer significantly shorter installation times compared to EAB. This difference reduces the duration of the surgical procedure, which also decreases operating costs and improves biosecurity by minimizing risks associated with the use of wires. While EAB remains a valid option for IMF, screw methods, such as IMFS and HAB, present significant advantages in terms of time, biosecurity, control of oral hygiene, and, in some cases, operating costs. However, the choice of method should be based on the clinical characteristics of the patient and the experience of the surgical team.

## Figures and Tables

**Table 1:** Characteristics of the results.

Author/Year	X	Age	FIM	Instalation time (minutes)	Wire gauge	Screw (Number/Size)	Occlusal stability	Tolerance	OH	Cost	Glove perforation	Root damage	Screw issue
King2019	69	31	EAB (A)HAB (B)	A>B31(A), 7(B)	24 Gauge	5 screws	-	-	A:B	-	A>B	0	-
Venugopalan 2020	32	46	EAB (A)HAB (B)	A>B76(A), 21(B)	26 Gauge	4 screws1.5x6mm	A:B	B>A	B>A	-	-	-	1(Group B)
Hamid2021	20	28	EAB (A)HAB (B)	A>B61(A), 41(B)	26 Gauge	4 screws2x6, 2x8mm	A:B	-	B>A	-	A>B	-	34% (Group B)
Sankar2023	44	31.5	EAB (A)HAB (B)	A>B82(A), 56(B)	26 Gauge	6 screws2x6, 2x 8,2 x10mm	A:B	-	B>A	B>A	A>B	2 (Group B)	2 (Group B)
Fernandes2023	28	29	EAB (A)IMF (B)	A>B43(A), 11(B)	-	4 screws2x7, 2x9mm	A:B	A:B	B>A	-	A>B	-	2 (Group B)
Merna2023	20	34	EAB (A)HAB (B)	-	26 Gauge	4 screws1.5x7mm	A:B	A>B	B>A	-	A>B	-	-
Pathak 2019	20	39	EAB (A)HAB (B)	A>B82(A), 27(B)	26 Gauge	4 screws1.5x8mm	A>B	B>A	A>B	-	-	-	-
Edmunds2019	93	28	EAB (A)IMF (C)HAB (B)	A>B>C98(A), 56(B), 48©	-	4 screws	-	-	-	A>B>C	B>C>A	0	B>C
Khelemsky 2019	102	18	EAB (A)HAB (B)	A>B186(A), 135(B)	-	-	-	-	-	A>B	-	-	-
